# Impact of patient-centered communication on patient satisfaction scores in patients with chronic life-limiting illnesses: an experience from Kenya

**DOI:** 10.3389/fmed.2024.1290907

**Published:** 2024-03-22

**Authors:** Betty Sirera, Violet Naanyu, Peter Kussin, David Lagat

**Affiliations:** ^1^Department of Internal Medicine, Moi Teaching and Referral Hospital, Eldoret, Kenya; ^2^School of Arts and Social Sciences, Moi University, Eldoret, Kenya; ^3^School of Medicine, Duke University, Durham, NC, United States; ^4^Department of Medicine, Moi University School of Medicine, Eldoret, Kenya

**Keywords:** patient-centered communication, patient satisfaction, chronic life-limiting illness, patient-doctor interaction, sub-Saharan Africa

## Abstract

**Background:**

Patient satisfaction remains a key area of interest worldwide; utilizing a patient-centered communication approach, particularly with patients with chronic life-limiting illnesses may be one way to achieve this. However, there is a dearth of empirical information on the effect of patient-centered communication strategies in patients with chronic life-limiting illnesses in Kenya on patient satisfaction.

**Objectives:**

The objective of this study was to assess the impact of patient-centered communication on patient satisfaction.

**Methods:**

We conducted our study at a tertiary teaching and referral hospital in Kenya. We utilized a quasi-experimental pre-test post-test study design and engaged 301 adult medical in-patients with chronic life limiting conditions. We randomized them to receive patient-centered communication, and evaluated the change in patient satisfaction scores using an adapted Medical Interview satisfaction Scale 21 (MISS 21).

**Results:**

Two hundred and seventy-eight out of 301 recruited participants completed the study. The baseline characteristics of the participants randomized to the control and intervention arms were similar. Although both the control and intervention arms had a decline in the mean difference scores, the intervention arm recorded a larger decline, −15.04 (−20.6, −9.47) compared to −7.87 (−13.63, −2.12), with a statistically significant mean difference between the two groups at −7.16 (−9.67, −4.46). Participants in the intervention arm were less likely to: understand the cause of their illness (*p* < 0.001), understand aspects of their illness (*p* < 0.001), understand the management plan (*p* < 0.001), receive all the relevant information on their health (*p* < 0.001), and to receive adequate self-care information (*p* < 0.001). They were also less likely to acknowledge a good interpersonal relationship with the healthcare providers (*p* < 0.001), to feel comfortable discussing private issues (*p* < 0.004), and to feel that the consultation time was adequate (*p* < 0.001).

**Conclusion and recommendation:**

Contrary to expectation, patient-centered communication did not result in improved patient satisfaction scores. Further studies can evaluate factors affecting and explaining this relationship and assess intermediate and long-term effects of provision of a patient-centered communication in diverse global contexts.

## Introduction

Patient satisfaction is how closely the patient’s vision of ideal care matches the perceptions of the care received (1). Patient satisfaction is the outcome measure which best evaluates the overall provision of quality healthcare (2) and it also provides important insights for healthcare professionals and health managers on health-seeking behavior and retention in care (3). This helps us to focus on the patients’ needs as well as to develop strategies to improve the quality of care provided.

One way of improving patient satisfaction is by viewing the patient as the center of care- an important stakeholder and a collaborator in good health outcomes with the right to make important decisions about the services rendered based on an informed understanding of their condition, treatment options and possible outcomes. This patient-centered approach considers the patient’s needs, views and preferences in the care provided (1). While the concept of patient-centered care is not new, there has been an increasing interest in its utilization the last decade to ensure that the care provided (i) aligns with the patient’s preferences and values and (ii) results in better quality and more cost effective care (1, 4). Fostering a patient-centered approach remains a highly desirable component of providing high-quality healthcare that is associated with greater patient satisfaction. Higher patient satisfaction rates have been associated with better retention to care, better adherence to management plans, and loyalty toward the institution and the doctor (1, 2).

A key component of this approach is effective provider-patient communication with respect for autonomy in the entire decision-making process (5). Several studies have illustrated the relationship between communication between patients and healthcare providers and patient satisfaction; patients who report good communication with healthcare providers trust the healthcare providers more and report better satisfaction with the healthcare process (6, 7). Although patient satisfaction is influenced by other factors such as infrastructure, hospital processes and clinical outcomes, a key area driving dissatisfaction is the doctor-patient interaction. In one study, 41% of respondents identified deficits in communication as a driver of dissatisfaction (8). This and other regional studies recommend utilizing a patient-centered communication strategy that allows better communication between patients and their healthcare providers and integrates patient involvement in the care process as a way to improve patient satisfaction (8–10).

Several strategies can be utilized to achieve patient-centered communication in a structured and empathetic manner. These include the SPIKES protocol, the Ask-Tell-Ask and the REMAP protocol. SPIKES is an acronym for a six-step sequence where S stands for setting, P for perception, I for invitation or information, K for knowledge, E for empathy, and S for summarize or strategize. This protocol involves providing the appropriate environmental setting for conducting healthcare discussions, evaluating the patient’s understanding of the disease and anticipated prognosis, obtaining an invitation to provide further information, addressing any emotion portrayed, and an opportunity to summarize the discussion points (11, 12). The Ask-Tell-Ask approach allows the healthcare practitioner to establish the patients and care-givers’ perception on the diagnosis made, provide them information, and establish the level of understanding (13). Finally, the REMAP stands for reframe, expect emotion, map out values, align with your patients’ values/goals, and propose a plan. This is a five step approach that provides an opportunity to contextualize the clinical state and provides an opportunity for further discussion and mapping out of further management plans based on the patient’s goals and values (14). The SPIKES protocol has been widely used in various clinical contexts including in Low and Middle income countries (LMIC). This approach ensures both the process and content of good communication in the interaction between the patient and healthcare providers and allows the patient to decide what information they receive.

The impact of communication strategies on patient satisfaction is assessed using various qualitative and quantitative approaches. Tools evaluating key performance indicators on patient satisfaction such as Picker Patient Experience Questionnaire (PSQ-18), the consumer assessment health plans (CAHPS), and the Functional Assessment of Chronic illness Therapy (FACT) satisfaction tools (15) include sections on communication between patients and their providers. The Medical Interview Satisfaction Scale (MISS-21), is a 21-item questionnaire that focusses primarily on the processes and content of good communication and its impact on patient satisfaction. The adapted version has been used in sub-Saharan Africa with good construct validity and internal consistency (16, 17).

Data on the effect of patient-centered communication on patient is derived from studies conducted in High Income and western countries and there is a paucity of evidence that this approach will result in increased patient satisfaction in Kenya. Therefore, the objective of this study was to assess the impact of patient-centered communication on patient satisfaction in patients admitted with chronic life-limiting illnesses in a tertiary teaching and referral hospital in Kenya.

## Methodology

### Study design

We utilized a quasi-experimental pretest- post-test design with randomized allocation to the control and intervention arms of the study arms to evaluate the impact of a patient-centered communication strategy on patient satisfaction among patients with chronic life-limiting illnesses.

The control arm of the study received standard of care with discussion of the patients’ clinical conditions driven by the healthcare providers and the content of the information shared at the discretion of the individual healthcare provider. Additionally consultation with the palliative care team, which utilizes a patient-centered communication approach, was also available at the discretion of the healthcare providers.

The intervention arm received patient-centered communication through goals of care discussions. An intervention discussion guide (Supplementary Appendix 7) was used to ensure the following six domains were included: a discussion on the documented clinical diagnosis to ensure the participants understood their diagnosis; the results of investigations done; the proposed management plan; the discussion that the anticipated illness trajectory was a chronic one; review of the participant’s needs and concerns; and the role the participants wanted their families to play in their care and associated care plans.

This intervention involved two sessions and both sessions were conducted during the in-patient stay of the participants. The first of the two sessions covered the six discussion domains provided above, while the second session was used to address any new concerns and provide an opportunity to address any follow-up questions.

The strategy used for patient-centered communication during the intervention was the SPIKES protocol that involves setting up the scene for the discussion; establishing the patients perception of their medical condition; obtaining and invitation to start a discussion; sharing knowledge with the patient and empathetically addressing emotion; and finally summarizing the discussion and strategizing or planning next steps (12).

### Study context and location

The study location was a 194-bed, general inpatient medical ward at a public tertiary referral hospital in Kenya.

### Study population

We recruited patients older than 18 years of age admitted to the inpatient medical service between August and December 2019 with a documented diagnosis of a chronic life-limiting illness as identified by the Supportive and Palliative Care Indicator tool (SPICT™)^1^. This included patients with end stage renal disease who were not kidney transplant candidates, patients with advanced heart failure, complicated and advanced human immunodeficiency virus (HIV), chronic respiratory failure and chronic liver failure. We used a Karnofsky performance score higher than 30 as the cut-off to identify individuals with chronic life-limiting illnesses who were at minimum, partially capable of participating in activities of self-care (Supplementary Appendix 4).

We excluded patients with advanced cancer, as a patient-centered communication approach, through goals of care discussions, is the recommended standard of care for this group of patients according to the Kenya National Cancer treatment protocol 2019. Patients with impaired cognition as determined using the six-item cognitive impairment test (6CIT) (Supplementary Appendix 3) and those unable to converse in English or ‘spoken Kiswahili’ were excluded.

### Sample size calculation

The sample size was calculated to compare the proportion of patients with good post-test satisfaction scores (≥4) between the two study groups. The calculated sample size of 255 participants was adjusted for a 20% attrition rate to a total of 306 participants. This provided adequate power to detect an intervention effect was used. By using a 1:1 allocation ratio, we anticipated the total number of participants per arm to be 153. We hypothesized that the participants receiving patient-centered communication, i.e., those in the intervention arm would have a higher proportion of participants with good post-test satisfaction scores compared to those in the control arm.

### Study variables and measures

The study outcome was the perceived patient satisfaction with the patient-healthcare provider communication as evaluated using an interviewer-administered adapted version of the Medical Interview Satisfaction Scale 21 (MISS 21) (Supplementary Appendix 6). We chose this tool as it considers both the processes and contents of good communication in the healthcare setting. It evaluates four key elements: information provision (four items), communication skills (nine items), confidence in the doctor (six items) and consultation time (one item) on a five-point Likert scale of responses.

The questions in the communication skills subsection of the MISS-21 questionnaire focuses on the components, both style and content, that are major determinants of good communication with healthcare providers. These include establishing rapport, provision of adequate health-associated information, and the consideration of the patient’s perspective, appreciation of the patient’s feelings and showing empathy, active listening and maintaining respect throughout the whole process (18).

The information provision section of the MISS-21 tool evaluates the effect of four components of information provision on patient satisfaction scores: understanding of the diagnosis and test results, understanding of the management plans, and provision of information on self-care and provision of all health information deemed necessary by the patient.

The patients’ confidence section assesses the participants’ perception of the treatment plan given, the advice given by healthcare providers and the ability of the healthcare providers to relieve the concerns of the participants on their illness. It also assesses the participants feeling of ease when communicating with the healthcare providers, ease of the participants to discuss personal information and the participants’ perception that their privacy was considered during the interaction.

The responses in the adapted questionnaire are scored between one and five, where a score of five corresponds to the most positive responses. To avoid confusion, a score of five connoted strong agreement and a score of one corresponded to strong disagreement with the phrase given. For each respondent, the overall score was the sum of all the 21 choices. For each item, we considered a score of four or more to correspond to satisfaction.

We evaluated the face validity and content validity of this questionnaire before the start of data collection by running a pilot test with 15 patients from the outpatient department of the hospital with chronic life-limiting illnesses. Following this, we adjusted the study tool by rewording the phrases and adjusting the order of the questions to ensure a good flow of the items during the filling of the questionnaire.

In addition to the adapted version of the Medical Interview Satisfaction Scale 21 (MISS 21), demographic data such as the age, gender, level of education, employment status/occupation and marital status and documented clinical diagnosis on all enrolled patients was also obtained.

The adapted MISS-21 questionnaires was administered twice (pre-test and post-test) for each participant in the control and the intervention group, with the patient-centered communication intervention being done in between for the intervention group. We conducted the post-test questionnaire administration on average, 10 days after the pre-test and before hospital discharge.

### Enrolment procedure

At the beginning of the study, the research team obtained formal permission to access patients’ identifying data for use in this research. The research team recorded names of all new patients admitted to the medical ward. One trained research assistant approached the patients to obtain informed consent and screened to identify patients who met the study’s inclusion criteria. This included those with documented diagnosis of a chronic life-limiting illness as identified by the Supportive and Palliative Care Indicator tool (SPICT™) (Supplementary Appendix 3), a Karnofsky performance score higher than 30 (Supplementary Appendix 4) and without significant cognitive impairment as determined using the six-item cognitive impairment test (6CIT) (Supplementary Appendix 3). We entered this information into a form capturing the patient’s demographic data and diagnosis. This form also contained a section to allow for documentation of the reason for the ineligibility of potential participants. The patient’s hospital registration number was included in the screening form to allow for easier identification of the patient’s file in case clarifications were required and for the process of documentation of any recommendations made.

The participants who met the eligibility criteria and gave consent were given a unique personal identifier/number. They then selected a sealed envelope with a random number with a 1:1 individual randomization strategy generated using the RAND function in the Microsoft Excel program prior to the start of the study. Participants with odd random numbers were in the control arm and those with even numbers were in the intervention arm of the study. A list containing the name and bed number of the participants in the intervention arm was provided to the research team members administering the intervention each day to facilitate the planning and implementation of the intervention.

### Recruitment schema

**Figure 1:**
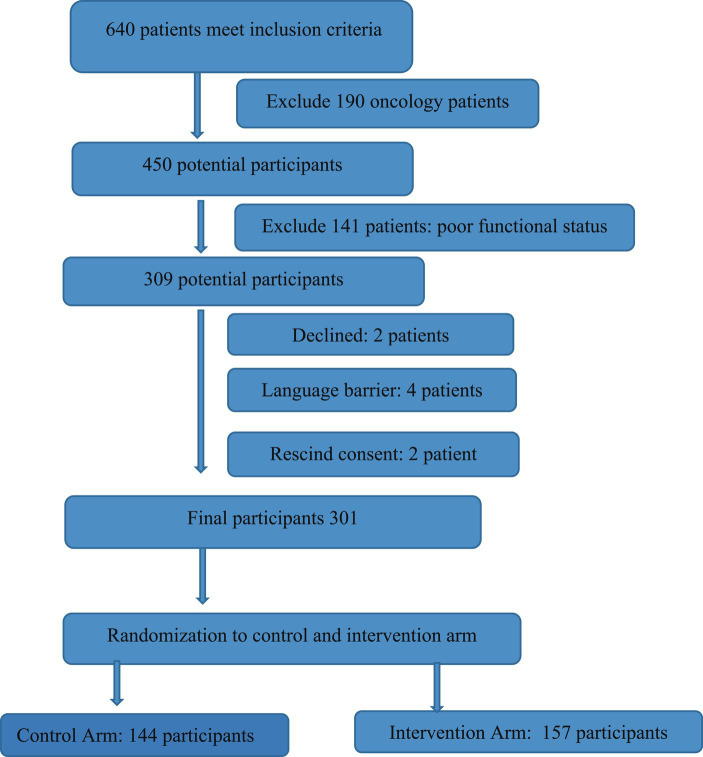
Recruitment schema.

From the beginning of August to the end of December 2019, we approached 1,240 patients of whom 640 met the inclusion criteria. We recruited 301 participants in the study, with the anticipated recruitment of a 306 participants; this was 98% of the calculated sample size with attrition factored. Out of the 301 participants, 278 participants completed both phases of the study (pre-test and post-test) which comprises 91% of the initial anticipated participants. This was adequate to address study objectives. The Figure 1 summarizes the recruitment process of the study.

### Data collection and handling

Two trained research assistants administered both the adapted MISS-21 pre-test and post-test questionnaires from the beginning of August to the end of December 2019. The questionnaires were administered in English and “conversational Kiswahili” at the wellness tent or by the bedside for participants confined to bed. The “wellness tent” is a semi-permanent structure located within the medical ward complex that is used by the healthcare providers in the inpatient medical ward to provide a private and comfortable space for patient sessions such as counseling session and family conferences.

We collected and directly entered the demographic data, clinical diagnosis and the results of the MISS-21 questionnaire into REDCapR, a web-based application used to record data from research for ease of data cleaning and handling. At the end of the study, we exported the results to Microsoft Excel for analysis. The average duration for the completion of the MISS-21 questionnaire was 35–40 min.

### Intervention administration

In preparation for this study, one of the research assistants, a registered clinical officer and a Master in psychology student and the primary researcher, a Masters of Internal medicine student, undertook an online training course with the Center to Advanced Palliative Care (CAPC) on the approach to patient-centered communication and ways to improve our communication skills. CAPC is an organization whose mission is to improve access to quality palliative care for patients with life-limiting illnesses by offering tools and training opportunities for healthcare workers all over the world. The research team members providing the intervention also worked alongside the palliative care providers at the hospital to gain some experience on approaches utilized in the hospital during patient evaluation and reviews.

Majority of the patient-centered communication interventions were held in the ‘wellness tent,’ a semi-permanent structure located within the medical ward complex. This site allowed for a private, comfortable and non-threatening setting, the ideal setting for complex conversations. For participants confined to their beds, we provided the intervention at the bedside during non-visiting hours. The participants were encouraged to invite members of their family or their caretaker for the discussions and all the participants in the intervention arm did this. On average, the first session took approximately 65–90 min and the follow-up session, done 24 h later for approximately 25–35 min.

Soon after the sessions, we documented the summaries of the discussions and any recommendations made in the patient’s chart and directly communicated to the doctor in the primary team managing the patient. We reviewed copies of this documentation in the analysis phase to assess for alignment with the protocol. For the majority of the participants, 86%, we were able to complete the two discussions fully.

For a proportion of the patients in the intervention group (36%), the primary team made further consultation with the palliative team for further discussion and planning follow-up in the outpatient setting and continued care. Only 7% of participants from the control arm received a consultation with the palliative care teams during the study period.

### Data analysis

Out of the 301 recruited participants, 23 participants had missing data sets. From the intervention group, one participant did not have both the pre-test and post-test results and 10 participants did not have post-test results while 13 participants in the control group did not have the post-test results. The participants with the missing post-test data either were discharged home before the completion of the study (3 participants), transferred to critical care units (3 participants) or died (17 participants) before the post-test was administered.

For the missing post-test variables (6.97%), we used data missing completely at random approach at analysis, given that the proportion of missing data was small and we had adjusted for it during the sample calculation.

The data was analyzed using the R-statistical software package on an intention-to-treat protocol. We summarize the categorical variables as percentages and then compared by the different treatment groups in tables and charts for ease of presentation and interpretation.

Chi-squared and Fischer exact test of significance was used to determine relationships between the categorical variables with a level of significance at *p* < 0.05.

The outcome of the study was defined as the mean difference between the post-test overall score and the pre-test overall score of the MISS-21 questionnaire. The overall change was calculated as a function of the mean difference score by subtracting the total post-test scores from the total pre-test score. As this was a quasi-experimental study, the unadjusted (no control for the potential confounders) and adjusted (controlling for age, sex, education level, marital status and performance status of the participants) mean difference scores were also calculated.

As the questions in the MISS-21 are grouped into patients’ confidence in the doctor (six items), communication skills (nine items) and information provision (four items); we calculated the unadjusted and adjusted change in score for each of these categories for both the pretest and posttest scores.

ANOVA models were used to test for differences between continuous variables in the intervention and control groups while the Fisher exact test was used to perform the same test in categorical variables. *p-*values for these tests were evaluated at <0.05 to be statistically significant.

Analysis of covariance (ANCOVA) regression method was used to test for the mean difference in the MISS-21 scores for the control and intervention arms of the study and a 95% confidence interval was used to assess for statistical significance. The dependent variable in the regression was the change in the scores, where potential confounders such as age, sex, level of education, marital status, and the pretest scores were also adjusted for in the analysis.

### Ethical considerations

The Moi University/MTRH-Institutional Research and Ethics Committee (IREC) approved this study with the approval number FAN: IREC 3228, permission to conduct the study was obtained from the hospital administration. All the participants of the study gave informed consent before participation in the study. Any concerns raised by the participants in the study and any recommendations made were discussed with the primary team managing the patient.

## Results

### Baseline characteristics of the participants

The key baseline characteristics of the participants in the intervention and the control group were largely similar. Table 1 summarizes the baseline characteristics of the participants. The mean age of the participants was 50.9 years with a predominance of male participants (57.8%). The majority of the participants, 58.8% had a low education level with either no education (23.6%) or incomplete primary (34.6%) and were married, 181 (60.1%). As such, age, gender, education and marital status were not significantly associated with randomization to the control or intervention group.

**Table 1:** Characteristics of trial study participants.

	Total 301	Control 144 (48%)	Treatment 157 (52%)	*P-*values
Age				
Mean (*sd*)	50.9 (17.6)	50.2 (17.4)	51.6 (17.8)	0.504^*^
Gender				
Female	127 (42.2%)	59 (41%)	68 (43.3%)	0.727^@^
Male	174 (57.8%)	85 (59%)	89 (56.7%)	
Education				
Tertiary education	28 (9.3%)	15 (10.4%)	13 (8.3%)	0.951
Completed secondary education	31 (10.3%)	15 (10.4%)	16 (10.2%)	
Completed primary education	67 (22.3%)	30 (20.8%)	37 (23.6%)	
Incomplete primary education	104 (34.6%)	51 (35.4%)	53 (33.8%)	
No schooling	71 (23.6%)	33 (22.9%)	38 (24.2%)	
Marital status				
Divorced	3 (1%)	2 (1.4%)	1 (0.6%)	0.087
Married	181 (60.1%)	82 (56.9%)	99 (63.1%)	
Separated	11 (3.7%)	8 (5.6%)	3 (1.9%)	
Single	59 (19.6%)	34 (23.6%)	25 (15.9%)	
Widowed	47 (15.6%)	18 (12.5%)	29 (18.5%)	
Karnofsky index				
20	1 (0.3%)	0 (0%)	1 (0.6%)	0.718
40	44 (14.6%)	25 (17.4%)	19 (12.1%)	
50	155 (51.5%)	72 (50%)	83 (52.9%)	
60	83 (27.6%)	38 (26.4%)	45 (28.7%)	
70	15 (5%)	7 (4.9%)	8 (5.1%)	
80	3 (1%)	2 (1.4%)	1 (0.6%)	

^*^ANOVA test, ^@^Fisher exact test.

The participants recruited in the study had a wide range of chronic life-limiting conditions. The most common conditions were: end-stage renal disease on hemodialysis (28%), advanced HIV with clinical stage 4 disease (21%), advanced heart failure with poor functional status (17%), and advanced chronic respiratory disease (21%).

Table 2 summarizes the distribution of the various conditions among the participants in the two groups. The distribution of the various life-limiting conditions of the participants recruited and randomized to the intervention and control groups was fairly matched.

**Table 2:** Diagnosis distribution among the participants.

Diagnosis	Total (*n* = 301)	Control (*n* = 144)	Intervention (*n* = 157)
End-stage renal disease	85 (28.2%)	38 (26.4%)	47 (29.9%)
Chronic respiratory disease with complication	63 (20.9%)	27(18.8%)	36 (22.9%)
Complicated HIV	63 (20.9%)	33 (22.9%)	30 (19.1%)
Heart failure	52 (17.2%)	26 (18.1%)	26 (16.6%)
Chronic liver disease	19 (6.3%)	9 (6.3%)	10 (6.4%)
Neurological disorder	17 (5.64%)	10 (6.9%)	7 (4.5%)
Others	2 (0.66%)	1 (0.7%)	1 (0.6%)

### Estimate of the intervention effect on the outcomes measured

#### Overall satisfaction score

**Figure 2:**
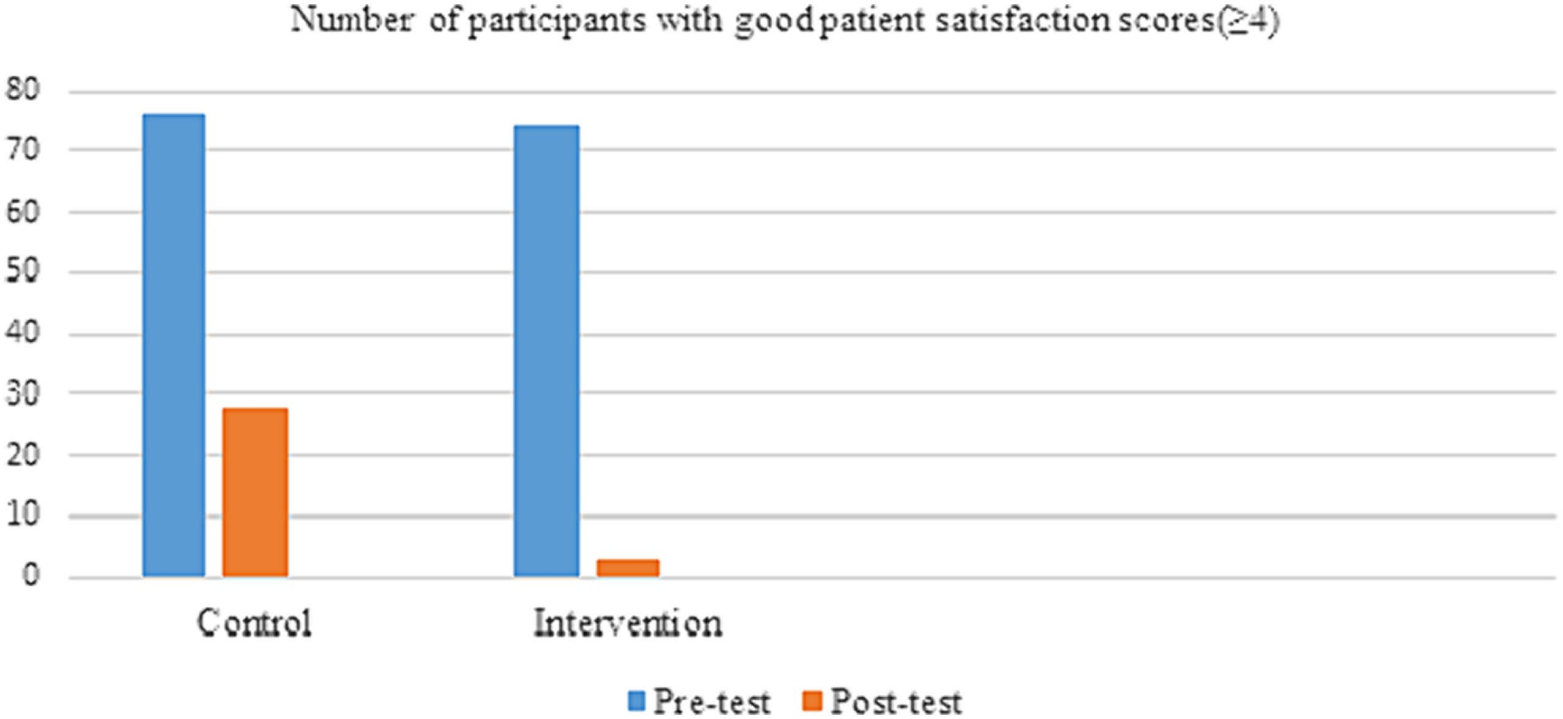
Change in the number of participants with an overall satisfaction score of ≥4.

The overall proportion of satisfied participants (mean score ≥ 4) at baseline as evaluated by the pre-test scores was found to be at 50%, with 76 (52.7%) participants in the control and 74 (47.3%) participants in the intervention arms with good satisfaction scores. The calculated Fisher’s Exact test for the two groups was calculated at 0.4186 which was not significant at a 95% confidence interval and the Odds ratio was 0.80 (0.50–1.30).There was a considerable reduction in overall patient satisfaction at the point of the administration of the post-test interview in both groups but worse in the intervention arm of the study. The participants with good satisfaction scores reduced to 28 (21.4%) participants in the control arm and 3 (2.04%) participants in the intervention arm (Figure 2). The Fisher’s Exact Test was calculated at a value of <0.00001 at a 95% confidence interval that is statistically significant. The calculated Odds ratio was 0.08 (0.02–0.26) suggesting a 92% chance of less satisfaction in the intervention arm compared to the control arm.

#### Estimate of the intervention effect on the mean overall change in scores

In both the control and intervention groups, there was a decline in the overall scores, as shown in Table 3, with the control group reporting an adjusted score of −7.87 (−13.63, −2.12) while the intervention group reporting a greater decline of −15.04 (−20.6, −9.47). The difference between the intervention and control group was a decline of −7.16 (−9.67, −4.66) points, and based on the confidence interval that excludes zero, this was a statistically significant effect change.

**Table 3:** Overall change in MISS-21 scores.

	*N*	Control group	Treatment group	Treat – control
Unadjusted change score	278	−9.9 (−12.41, −7.39)	−18.65 (−21.01, −16.28)	−8.75 (−12.19, −5.3)
Adjusted change score	278	−7.87 (−13.63, −2.12)	−15.04 (−20.6, −9.47)	−7.16 (−9.67, −4.66)

#### MISS-21 individual item analysis

When evaluating the single items under the three main sub-items of patient confidence, information provision and patient confidence scores, it was noted that several individual items had statistical significance. Under the communication skill scores, two out of the nine items were found to have statistical significance with participants in the intervention arm being less likely to: understand the explanation given on the cause of ill health (*p* < 0.001) and to perceive that the healthcare provider was interested in more than just disease process (*p* < 0.001). Under the information provision score items, all four questions in this subsection were statistically significant. The participants in the intervention arm were less likely to feel that they; understood the cause of their illness (*p* < 0.001), understood all aspects of their illness (*p* < 0.001), understood their management plan (*p* < 0.001), had received all the relevant information on their health (*p* < 0.001), or had received adequate self-care information (*p* < 0.001). Under the patient confidence subset, only one out of the six questions was statistically significant; with the participants in the intervention arm more likely to report the participants’ feeling uncomfortable discussing private issues with the healthcare providers (*p* < 0.004).

On the two individual questions, patients in the intervention group were less likely to feel that the consultation time was adequate (*p* < 0.001) and less likely to have overall patient satisfaction (*p* < 0.001) and both were found to be statistically significant.

Table 4 summarizes the results of the single items of the MISS-21 tool.

**Table 4:** Analysis of single item pre- and post-test of the 21 Miss Likert Questionnaire.

Variable name		Total	Control	Intervention	*P-*value
Communication skills					
The doctor greeted me before addressing my complaints (CS)	Pre-test	1.5 (0.8)	1.5 (0.9)	1.5 (0.7)	0.559
	Post-test	1.2 (0.4)	1.2 (0.4)	1.2 (0.4)	0.44
The doctor explained the cause of my ill health well (CS)	Pre-test	3.4 (1.4)	3.4 (1.4)	3.5 (1.3)	0.587
	Post-test	2.2 (1.1)	2.6 (1.3)	1.8 (0.7)	<0.001
The doctor gave me a chance to say or ask all I wanted (CS)	Pre-test	3 (1.3)	2.9 (1.4)	3.1 (1.3)	0.139
	Post-test	1.9 (0.9)	2.1 (1)	1.8 (0.7)	0.006
The doctor listened patiently to me (CS)	Pre-test	2.2 (1.1)	2.1 (1.1)	2.3 (1.1)	0.181
	Post-test	1.7 (0.6)	1.8 (0.8)	1.6 (0.5)	0.007
The doctor did not ignore any of the things I said or the complaints I had (CS)	Pre-test	2.6 (1.3)	2.5 (1.3)	2.8 (1.4)	0.08
	Post-test	1.8 (0.9)	2 (1)	1.7 (0.8)	0.013
The doctor did not use any words that I did not understand (CS)	Pre-test	2 (1)	1.9 (1)	2.1 (1)	0.035
	Post-test	1.6 (0.6)	1.6 (0.6)	1.6 (0.6)	0.402
The doctor seemed interested in me as a person and not just my illness (CS)	Pre-test	2.4 (1.1)	2.3 (1)	2.4 (1.1)	0.571
	Post-test	1.7 (0.8)	1.9 (0.9)	1.6 (0.5)	<0.001
The doctor spoke politely to me (CS)	Pre-test	1.8 (0.8)	1.8 (0.8)	1.8 (0.9)	0.73
	Post-test	1.6 (0.5)	1.6 (0.5)	1.5 (0.5)	0.575
The doctor was warm and empathetic to me (CS)	Pre-test	2.1 (1)	2.1 (1.1)	2.1 (1)	0.714
	Post-test	1.6 (0.6)	1.6 (0.6)	1.6 (0.5)	0.413
Information provision					
I understood what the doctor wants us to do to manage my condition (IP)	Pre-test	2.9 (1.2)	2.8 (1.2)	2.9 (1.2)	0.465
	Post-test	2 (1)	2.3 (1.2)	1.8 (0.7)	<0.001
The doctor gave me all the information I was expecting to receive about my health (IP)	Pre-test	3.3 (1.2)	3.2 (1.3)	3.4 (1.1)	0.239
	Post-test	2.2 (1)	2.5 (1.2)	1.9 (0.8)	<0.001
I understand my illness (diagnosis, test results and treatment plan) better after talking to the doctor (IP)	Pre-test	3.3 (1.3)	3.2 (1.3)	3.4 (1.2)	0.229
	Post-test	2.1 (1.1)	2.4 (1.2)	1.8 (0.8)	<0.001
The doctor told me how to care for myself given my condition (IP)	Pre-test	3.2 (1.3)	3.1 (1.4)	3.2 (1.3)	0.729
	Post-test	2.1 (1.1)	2.4 (1.3)	1.8 (0.8)	<0.001
Patient’s confidence					
The doctor seemed to know what to do about my problem (PC)	Pre-test	2.2 (0.9)	2.1 (0.9)	2.2 (0.9)	0.652
	Post-test	1.7 (0.7)	1.8 (0.8)	1.6 (0.6)	0.064
I think the doctor’s advice is appropriate for my situation (PC)	Pre-test	2.7 (1.2)	2.6 (1.1)	2.8 (1.2)	0.201
	Post-test	2.1 (0.9)	2.2 (1)	2 (0.9)	0.133
The doctor relieved my worries about my illness (PC)	Pre-test	3.5 (1.4)	3.4 (1.4)	3.5 (1.3)	0.458
	Post-test	2.7 (1.3)	2.8 (1.4)	2.5 (1.3)	0.027
I felt comfortable talking to the doctor (PC)	Pre-test	2.2 (1)	2.2 (1)	2.3 (1.1)	0.401
	Post-test	1.7 (0.6)	1.8 (0.7)	1.6 (0.5)	0.009
I could talk freely to the doctor about my private issues (PC)	Pre-test	2.4 (1.2)	2.4 (1.2)	2.4 (1.2)	0.816
	Post-Test	1.7 (0.7)	1.9 (0.8)	1.6 (0.6)	0.004
The doctor paid enough attention to my privacy (PC)	Pre-test	2 (1)	2 (1)	2.1 (0.9)	0.737
	Post-test	1.7 (0.6)	1.7 (0.6)	1.6 (0.6)	0.591
Doctor’s time					
I had enough time with the doctor (CT)	Pre-test	2.3 (1.1)	2.2 (1.2)	2.4 (1.1)	0.334
	Post-test	1.7 (0.8)	1.9 (0.9)	1.6 (0.6)	0.001
Overall satisfaction					
All things considered, I am satisfied with the interaction between the doctor and I	Pre-test	2.7 (1.1)	2.7 (1.1)	2.7 (1.1)	0.758
	Post-test	1.8 (0.7)	2 (0.8)	1.6 (0.5)	<0.001

## Discussion

To the best of our knowledge, this is the first study of its kind to evaluate the impact of patient-centered communication on patient satisfaction in the Kenya. At the start of the study, based on past studies (19–21), we thought the process of having comprehensive and candid discussions with the participants of the study would improve their perception of the patient-doctor interaction and therefore the perceived quality of care received. However, the findings of this study contradict the proposed hypothesis that patient-centered communication would result in improved patient satisfaction scores as the intervention arm performed worse compared to the control arm.

### Reduction in overall proportion of participants with good satisfaction scores

At baseline, 50% of the participants in both the control and intervention arm had adequate overall patient satisfaction with the doctor-patient interaction. However, the post-test evaluation demonstrated a considerable reduction in the proportion of satisfied participants in both arms.

The duration between base-line and end-line tests among patients can have implications for the outcomes observed (22) and may have contributed to this reduction in satisfaction scores. The duration between the pre-test and post-test evaluation for both the control and treatment arm was 10 days. As such, without an appropriate decay period between the pre-test and post-test administration, the outcome of the post-test results can be influenced by the administration of the pre-test due to the possible instructional effect of the pre-test, particularly if the participants remembered and reflected on the questions (23). This instructional effect can contribute to a response shift with a change of the participants’ standards of evaluation of satisfaction with their interaction with the healthcare providers through reevaluation of the standards of determining adequate satisfaction, adjustment in the values placed on various aspects of the interaction and redefinition of the adequate satisfaction (18, 24).

Patient-centered communication in this study worsened the participants’ satisfaction scores. Autonomy is a key consideration in patient-centered communication that allows the patients to receive information about their clinical condition that allows them to participate in shared decision making to varied degrees as determined by the patient (5, 25). However, there is some cultural difference in healthcare communication, particularly in patients with chronic life limiting or terminal medical conditions. Mcgrath et al. (26) demonstrated that open communication about chronic illness may be found ‘frightening’ in some cultures and a more indirect communication style may be preferred. In-fact, in particular cultural and social situations, a direct approach in communication with patients could be disadvantageous (27) and paternalistic or a mix between paternalistic and patient-centered communication may be ideal for some societies (28, 29) particularly in patients with insufficient health literacy.

In the next sections, we postulate various factors that may contribute to the reduction in patient satisfaction under the main subsections of the MISS 21 questionnaire.

### Communication skills sub-section

Under this subsection, the participants in the intervention arm were more likely to report inadequate satisfaction on the explanation on the cause of their illness (*p* < 0.001) and felt the health-care teams were not interested in the individual and were more focused on the illness (*p* < 0.001). This was an interesting and unexpected finding from this study; the focus of the intervention was to explain the participants’ chronic illness, particularly on the diagnosis and the available test results, in a way that would allow the participant to understand; we also provided an opportunity to answer any questions that arose. We also endeavored to individualize the discussions and to discuss the non-medical needs of the participants.

It has been noted that patients report being discontent with communication from healthcare providers even when the providers rate their interaction with the patient highly (30). Even when the correct terminology and appropriate information are given, the majority of patients do not identify the issues that were discussed (31). It is therefore imperative for healthcare providers to continue to assess and evaluate the communication to ensure that both parties have the same understanding.

### Information provision subsection

Regional and local studies report that information provision is a key element in improving patient satisfaction with the healthcare provided (17, 21). However, in this study, participants who received patient-centered communication focused on information provision were more likely to report dissatisfaction with the information about their diagnosis, test results, ongoing management plans and advice on self-care and reported the information provided did not meet their expectations with a *p*-value of <0.001 in all the questions.

Majority of the participants had low education levels with 58.8% of participants with either no or incomplete primary school education compared to only 19% of participants with completed secondary and tertiary education. Patients with low education levels have low health literacy levels and therefore lower ability to access and understand healthcare related information (32). Accordingly, patients who struggle to understand healthcare information do not interact effectively with the healthcare providers and this influences their satisfaction. It is worth noting that despite efforts by the research team to provide information in a manner understandable and relatable to the participants, the explanation of information particularly relating to the medical diagnosis, the investigations and the management plan is not available in ‘conversational Kiswahili’ and the local languages. This presented a challenge of making sure that the information conveyed was understandable to the participants, the majority of whom had lower educational levels and were not comfortable holding conversations in English. This challenge is not unique to this study. Many African populations use native languages, however English is the main language of instruction in medical education (33). As such, healthcare providers need to translate clinical and technical information and do code-switching between standard English, vernacular English, spoken Kiswahili and native languages during conversation with non-English speaking patients (34). The lack of medical information in native languages influences how health related information is conveyed and received by patients. It is therefore probable that this and low health literacy levels among our study participants affected their reception of the information.

Another important aspect to consider is the role of illness perception that influences the reception and interpretation of any health-associated information. Illness perceptions are cognitive beliefs or views that people have that help them cope with their illness. They include the illness identity (the name and symptoms of the illness), the causal component (the individual understanding of the cause of illness) and the consequence component (the consequence of the illness to the individual and family and the control that the individual has on the illness) (35). Illness perceptions in sub-Saharan Africa are negative in nature due to the physical, psychological, emotional, spiritual and economic consequences that they bear (36). Though the effect is varied, patients with negative illness perceptions are generally less receptive to information that is contrary to their beliefs (35). Reflectively, understanding that one has a chronic life-limiting illness may raise concerns of anticipated increased dependency on caregivers, concerns of potential actual and perceived stigma as well as thoughts of ‘unfinished business’ that needs to be addressed and this may cause anxiety for some patients and make them less receptive to the information provided.

Consideration should also be given to the possibility of the effect of information overload, which can be thought of as ‘too much’ information, not only in the amount but also in the intensity and complexity of the information, that makes it difficult to understand the issues(s) being discussed (37). The conversations with the participants in the intervention arm was done over a period of two sessions and the discussions can be considered loaded in nature in both the amount and the complexity of information particularly when discussed with laypersons. It is conceivable that, although the information provided and issues discussed were beneficial, these conversations may have resulted in an information overload with the result that the information became an obstacle rather than helpful as intended by the research team.

### Patient’s confidence subsection

Under this subsection, the participants in the intervention arm were less likely to feel comfortable when discussing personal or private issues with a *p*-value of 0.004. The lack of ease during discussions of personal matters is an indication of a poor interpersonal connection between the patient and the healthcare provider. Some patient factors may contribute to the acceptability of the formation of interpersonal connections and interpersonal connections may not be as highly valued in some cultures. In their study, Waweru et al. (38) found that interpersonal components of the patient-healthcare provider were not expected and a large number of the study’s participants did not expect to, nor openly discuss personal issues with their healthcare providers, as this was a role ascribed to closer social contacts such as family and elders. Additionally, the young age and the female gender of the primary investigator and research assistant who were involved in the administration of the intervention may have been a limitation in establishing a good interpersonal connection with the participants. Although this represents the current trend in healthcare providers in Kenya with a predominant young work force (39), we acknowledge that some participants particularly older and male participants may have been uncomfortable discussing some topics with younger and female healthcare providers. This is a limitation in this study and an area of improvement and a topic for future studies.

### Adequacy of the time spent with participants

Several studies, including a study from sub-Saharan Africa, show that patients with longer consultations and who spend longer communicating with healthcare practitioners report having higher satisfaction scores and that most patients report having shorter than ideal consultations time (16, 40). Although there is no established standard duration of consultations, longer durations of consultations are associated with more elaborate healthcare discussions and evaluation of psychosocial aspects of care (40).

On average, the intervention took 65–90 min for the first session and the follow-up session 25–35 min; this is considered a longer duration than most standard healthcare consultations. Despite this, participants in the intervention arm were more likely to report inadequate time with healthcare providers compared to the control arm. In their study, Ogden et al. (41) postulated that the perception of inadequate time with healthcare providers was associated with unmet needs particularly emotional needs rather than information giving. It appears that despite spending additional time with the participants in the intervention group, the time spent did not meet the needs of the participants and was thus not considered meaningful time.

## Conclusion

This study provides useful insight on possible impact of patient-centered communication on patient satisfaction in patients admitted with chronic life-limiting illnesses.

Surprisingly, in this study, provision of a patient-centered communication strategy did not result in improved patient satisfaction scores among patients with chronic life-limiting illnesses; the result was negative with lower patient satisfaction scores for the participants in the intervention group. The immediate effect of patient-centered communication strategies on patient satisfaction may not be linear in the Kenyan setting. Future studies ought to evaluate factors affecting and explaining this relationship. We ought to assess intermediate and long-term effects of provision of a patient-centered communication in diverse global contexts.

### Strengths and limitation

A strength of this study is its design that included intentional training of the field team to ensure a full understanding of the study and to enhance their communication skills. Due to the nature of the study and the structure of the study site, a public hospital general ward, we recruited participants from both arms from the same location and this could have resulted in contamination among participants. Furthermore, the intervention sessions were not audio recorded and were therefore not objectively assessed to check whether the content provided and processes of the discussions were adequate. These are potential areas of improvement in further studies.

## Data availability statement

The raw data supporting the conclusions of this article will be made available by the authors, without undue reservation.

## Ethics statement

The studies involving humans were approved by MTRH/MU-Institutional Research and Ethics Committee (IREC). The studies were conducted in accordance with the local legislation and institutional requirements. The participants provided their written informed consent to participate in this study.

## Author contributions

BS: Writing – review & editing, Writing – original draft, Visualization, Software, Resources, Project administration, Methodology, Investigation, Formal analysis, Data curation, Conceptualization. VN: Writing – review & editing, Validation, Supervision, Conceptualization. PK: Writing – review & editing, Validation, Supervision, Conceptualization. DL: Writing – review & editing, Supervision.

## Footnotes

^1^ https://www.spict.org.uk/
