# Supplementary material for: Impact of patient-centered communication on patient satisfaction scores in patients with chronic life-limiting illnesses: an experience from Kenya
Source: Front Med (Lausanne). 2024 Mar 22;11:1290907. doi: 10.3389/fmed.2024.1290907 (PMC10995390; doi:10.3389/fmed.2024.1290907)
Supplement: Supplementary file 1 [file Data_Sheet_1.docx]

**Impact of a patient-centred communication on patient satisfaction scores: an experience from Kenya.**

Sirera Betty^1*^, Naanyu Violet^2^, Kussin Peter^3^, Lagat David^1^

1. Department of Internal Medicine, Moi Teaching and Referral Hospital, Eldoret, Kenya.
2. School of Arts and Social Sciences, Moi University, Eldoret Kenya.
3. Duke University, School of Medicine, Durham, NC, USA

Correspondence

Sirera Betty

[sirerab@gmail.com](mailto:sirerab@gmail.com)

Appendix 1: Recruitment log

| **Date of hospital admission** | **IP No** | **Name** | **Date of birth** | **M/F** | **Hospital Bed No** | **File diagnosis** | **SPICT ^TM^-SA Criteria met** | **Karnofsky Perfomance status** | **6 CIT**  **score** | **Recruited**  **(reason for exclusion** |
| --- | --- | --- | --- | --- | --- | --- | --- | --- | --- | --- |
|  |  |  |  |  |  |  |  |  |  |  |
|  |  |  |  |  |  |  |  |  |  |  |
|  |  |  |  |  |  |  |  |  |  |  |
|  |  |  |  |  |  |  |  |  |  |  |
|  |  |  |  |  |  |  |  |  |  |  |
|  |  |  |  |  |  |  |  |  |  |  |
|  |  |  |  |  |  |  |  |  |  |  |
|  |  |  |  |  |  |  |  |  |  |  |
|  |  |  |  |  |  |  |  |  |  |  |
|  |  |  |  |  |  |  |  |  |  |  |
|  |  |  |  |  |  |  |  |  |  |  |
|  |  |  |  |  |  |  |  |  |  |  |
|  |  |  |  |  |  |  |  |  |  |  |
|  |  |  |  |  |  |  |  |  |  |  |
|  |  |  |  |  |  |  |  |  |  |  |

**Appendix 2: The six item cognitive impairment test (O’Sullivan et al., 2016)**

Instructions: please ask the patient the following questions and score within the range provided. Calculate the weighted score and calculate the total score. Participants with scores higher than 9 will be excluded from the study.

| \| **Test Question** \| **Score range** \| **Weighting** \| **Weighted score** \| \| --- \| --- \| --- \| --- \| \|  \|  \|  \|  \| \| What Year is it \| 0-1 \| x4 \|  \| \| What month is it \| 0-1 \| x3 \|  \| \| *Give the memory phrase e.g. (Peter/ Njoroge/ Uganda road/ Eldoret)* \|  \|  \|  \| \| About what time is it \| 0-1 \| x3 \|  \| \| Count back from 20-1 \| 0-2 \| x2 \|  \| \| Say months in reverse \| 0-2 \| x2 \|  \| \| Repeat the memory phrase \| 0-5 \| x2 \|  \| \| Total score for 6CIT \| 0-28 \|  \|  \| |
| --- | --- | --- | --- | --- | --- | --- | --- | --- | --- | --- | --- | --- | --- | --- | --- | --- | --- | --- | --- | --- | --- | --- | --- | --- | --- | --- | --- | --- | --- | --- | --- | --- | --- | --- | --- | --- | --- | --- | --- | --- |

###

**Appendix 3: The SPICT^TM^ tool for chronic illness.**

**Appendix 4: The Karnofsky performance scale index.**

The Karnofsky Performance Scale Index allows patients to be classified as to their functional impairment. The lower the Karnofsky score, the worse the survival for most serious illnesses

Instructions for use: select the rating of the patient based on the criteria based on brief evaluation on self-reported functional status.

| Rating | Criteria |
| --- | --- |
| 100 | Normal no complaints; no evidence of disease. |
| 90 | Able to carry on normal activity; minor signs or symptoms of disease. Able to carry on normal activity and to work; no special care needed. |
| 80 | Normal activity with effort; some signs or symptoms of disease. |
| 70 | Cares for self; unable to carry on normal activity or to do active work. |
| 60 | Requires occasional assistance, but is able to care for most of his personal needs. Unable to work; able to live at home and care for most personal needs; varying amount of assistance needed |
| 50 | Requires considerable assistance and frequent medical care. |
| 40 | Disabled; requires special care and assistance. |
| 30 | Severely disabled; hospital admission is indicated although death not imminent. |
| 20 | Very sick; hospital admission necessary; active supportive treatment necessary |
| 10 | Moribund; fatal processes progressing rapidly. Unable to care for self; requires equivalent of institutional or hospital care; disease may be progressing rapidly. |
| 0 | Dead |

**Appendix 5: Informed consent form (adapted from W.H.O consent form)**

**Study title: Effect of patient centred communication on patient satisfaction in patients with chronic life-limiting Illness at Moi Teaching and Referral Hospital.**

**This Informed Consent Form has two parts:**

- **Information Sheet (to share information about the research with you)**
- **Certificate of Consent (for signatures if you agree to take part)**

**You will be given a copy of the full Informed Consent Form**

**PART I: Information Sheet**

**Introduction**

We are doing research on the effect of discussions of management plans with patients and how this affects the level of satisfaction with the care provided. Information on the study will be provided to you and you will be invited to participate in the study. We will try and explain as much as possible to ensure you understand what will be involved in the study. If you do not understand anything as we go through the information, please feel free to ask me at any point. You will also be given time, if you require it, to decide whether you would like to be involved in the study.

**Description of the research and your participation**

There are many patients who have been unwell for a long time. I feel that the information provided to patients and their relatives may not be enough. This study is looking at how patients would feel about their care when provided with more detailed discussions on their illness.

Your participation in this research will involve interviews at two different times to evaluate how satisfied you are about your experience with the doctor. Some of the participants of this research will receive more targeted discussions in an attempt to see if this improves the perceived quality of care.

All patients admitted in the medical ward with a medical history of a chronic illness are invited to participate in the study. Your involvement in this research is completely on a voluntary basis: you are free to choose to participate in this study or not. Whether you choose or decline to participate in this study, your medical care will continue to be provided as usual. You are free to stop participating at any point of the study; this will also not affect the care you receive at this hospital.

**Risks and Benefits:**

There is no obvious risk in participating in this research. Any issues noted during the research process will be communicated to the doctors currently taking care of you. Your participation in this study may help in the future to improve the interaction between patients and their doctors and potentially the degree of patient satisfaction with care.

**Confidentiality:**

We will ensure that your right to privacy is respected at all times during the study. Your personal information will be kept private and only the researchers will have access to it. Your information will have a number instead of your name and all information collected will be stored safely in a lockable cabinet for the hard copies and in a password protected computed for the soft copies and will not be shared with anyone else.

**Utilization of the research findings:**

The findings of this research will be shared through meetings at the department of Internal Medicine, Moi University and also at a meeting at the School of Medicine Moi University; this meeting is open to members of the public. No confidential information will be shared in any forum for dissemination of the findings. The findings of this study will also be published to allow other people access to the findings of the research.

This document has been reviewed and approved by the institutional review and ethics committee, which is a committee responsible for making sure that any research being done is safe and that all participants involved in research are from harm.

You can ask me any more questions about any part of the research study, if you wish to. Do you have any questions?

**PART II: Certificate of Consent**

I have read/have been explained to the above information. I have had the opportunity to ask questions about it and any questions that I have asked have been answered to my satisfaction. I consent voluntarily to participate in this research.

Print Name of Participant__________________

Signature ___________________

Date ___________________________

Or

I have witnessed the accurate reading of the consent form to the potential participant, and the individual has had the opportunity to ask questions. I confirm that the individual has given consent freely.

Print name of witness_____________________ AND Thumb print of participant

Signature of witness ______________________

Date ________________________

**Statement by the researcher/person taking consent**

I have accurately read out the information sheet to the potential participant, and to the best of my ability made sure that the participant understands the research being undertaken.

I confirm that the participant was given an opportunity to ask questions about the study, and all the questions asked by the participant have been answered correctly and to the best of my ability. I confirm that the individual has not been coerced into giving consent, and the consent has been given freely and voluntarily.

 A copy of this consent form has been provided to the participant.

Name of person taking the consent________________________

Signature of Researcher /person taking the consent__________________________

Date ___________________________

Day/month/year

If you need further clarifications, feel free to contact IREC using the address below.

The Administrator IREC

Moi Teaching and Referral Hospital,

P.O Box 3, Eldoret.

Telephone number: 33471/2/3

##

### Appendix 6: Adapted medical interview satisfaction scale (MISS 21) tool

(Meakin & Weinman, 2002; Abioye Kuteyi et al., 2010)

**Satisfaction with doctor patient interaction at Moi Teaching and Referral Hospital medical ward.**

We are conducting a study to evaluate the level of satisfaction of patients admitted in the medical ward with regards to the doctor patient interaction. You will be asked to provide responses at the beginning and at end of your hospital admission.

Your responses will be kept confidential.

Instructions for administration: This is an interviewer administered questionnaire. Please read the questions out to the participants and give the options indicated.

**SECTION A**

| Personal identifier |  |
| --- | --- |
| Age |  |
| Sex |  |
| Level of education |  |
| Marital status |  |
| Occupation |  |
| Clinical Diagnosis |  |

**SECTION B**

Please state which response best describes your feeling toward your experience so far. We are interested in the response both positive and negative.

|  | Strongly agree | Agree | Uncertain | Disagree | Strongly  disagree |
| --- | --- | --- | --- | --- | --- |
| The doctor greeted me before addressing my complaints. (CS) | 5 | 4 | 3 | 2 | 1 |
| The doctor explained the cause of my ill health well. (CS) | 5 | 4 | 3 | 2 | 1 |
| The doctor gave me a chance to say or ask all I wanted (CS) | 5 | 4 | 3 | 2 | 1 |
| The doctor listened patiently to me(CS) | 5 | 4 | 3 | 2 | 1 |
| The doctor did not ignore any of the things I said or complaints I had. (CS) | 5 | 4 | 3 | 2 | 1 |
| I understood what the doctor wants us to do to manage my condition (IP) | 5 | 4 | 3 | 2 | 1 |
| The doctor gave me all the information I was expecting to receive about my health (IP) | 5 | 4 | 3 | 2 | 1 |
| The doctor did not use any words that I did not understand. (CS) | 5 | 4 | 3 | 2 | 1 |
| I understand my illness (diagnosis, test results and treatment plan) better after talking to the doctor. (IP) | 5 | 4 | 3 | 2 | 1 |
| The doctor seemed to know what to do for my problem. (PC) | 5 | 4 | 3 | 2 | 1 |
| I think the doctor’s advice is appropriate for my situation. (PC) | 5 | 4 | 3 | 2 | 1 |
| The doctor seemed interested in me as a person and not just my illness(CS) | 5 | 4 | 3 | 2 | 1 |
| The doctor told me how to care for myself given my condition (IP) | 5 | 4 | 3 | 2 | 1 |
| The doctor relieved my worries about my illness. (PC) | 5 | 4 | 3 | 2 | 1 |
| I felt comfortable talking to the doctor. (PC) | 5 | 4 | 3 | 2 | 1 |
| I could talk freely to the doctor about my private issues. (PC) | 5 | 4 | 3 | 2 | 1 |
| The doctor spoke politely to me. (CS) | 5 | 4 | 3 | 2 | 1 |
| The doctor was warm and empathetic to me (CS) | 5 | 4 | 3 | 2 | 1 |
| The doctor paid enough attention to my privacy. (PC) | 5 | 4 | 3 | 2 | 1 |
| I had enough time with the doctor (CT) | 5 | 4 | 3 | 2 | 1 |
| All things considered I am satisfied with the interaction between the doctor and I | 5 | 4 | 3 | 2 | 1 |

### Appendix 7: Patient centred communication check list (SPIKES Protocol approach)

**Set up the scene**

- prepare and get patient information before hand, discuss with relevant clinical teams.
- Ask Participant to invite people they would like to participate in the discussions and schedule a time.
- Give options of using the ward wellness tent for privacy.
- Introduce self and the participants available, provide information on the reason for conversation: I am doing my research to see if having targeted conversations with patient changes the doctor-health care provider relationship. This is my colleague and we will be speaking with you together. You have been selected to participate as we feel you might benefit from this interaction due to your ongoing medical issues. You had given consent earlier, are you still willing to participate in the study?

**Perception: Assess/Ask to establish patient understanding:**

‘What do you know about your illness?’

**Invitation: Obtain permission to provide more information.**

‘How much information would you like from me about your illness?’

**Knowledge sharing in non-technical terms.**

‘This is what my understanding of your illness is…’

Provide opportunity for clarification/ asking questions

**Allow and address Emotions of the participants and other members present.**

Give time for information to sink in.

Validate feelings expressed: Name the emotions, be understanding, respectful and supportive

**Summarise and strategize**

Check if Participant and the other people present are ready for more discussions. If not, defer to follow up sections.

Explore patient’s needs: ‘I hope that your symptoms will improve and that you will continue to live well for a long time. However, if things change, what are your most important goals if your health situation worsens?’

*Probes:

- goals for relationships with family and friends
- important life events or community activities in the future
- goals for the immediate family: support for the family
- emotional or psychological support/ counselling
- financial goals

‘What things worry you when you think about your future with your health?’

“What encourages you as you think about the future with your illness?”

“How much does your family know about your priorities and wishes?”

**conclude**

Summarise the main points of the discussion particularly the priorities.

Give opportunity for further follow up.
