# Supplementary material for: Impact of patient-centered communication on patient satisfaction scores in patients with chronic life-limiting illnesses: an experience from Kenya
Source: Front Med (Lausanne). 2024 Mar 22;11:1290907. doi: 10.3389/fmed.2024.1290907 (PMC10995390; doi:10.3389/fmed.2024.1290907)
Supplement: Supplementary file 2 [file Data_Sheet_2.pdf]

Section/Topic	Item No	Checklist item	Reported on page No**
**Title and abstract**	1a	Identification as a randomized trial in the title	No
	1b	Structured summary of trial design, methods, results, and conclusions (for specific guidance see CONSORT for abstracts)	Page 1, lines 86-106
**Introduction**			
Background and	2a	Scientific background and explanation of rationale	page 2
objectives	2b	Specific objectives or hypotheses	page 3 line 291-294
**Methods**			
Trial design	3a	Description of trial design (such as parallel, factorial) including allocation ratio	page 2, lines 220-224 and page 3, lines 285-290
	3b	Important changes to methods after trial commencement (such as eligibility criteria), with reasons	N/A
Participants	4a	Eligibility criteria for participants	Page 3,lines 264-282
	4b	Settings and locations where the data were collected	Page 3 , lines 258-259, page 4, lines 408-414 and line 438-433
Interventions	5	The interventions for each group with sufficient details to allow replication, including how and when they were actually administered	page 3 lines 255-253, Page 4 lines 423-453
Outcomes	6a	Completely defined pre-specified primary and secondary outcome measures, including how and when they were assessed	Page 3 lines 299-307, Page 4, lines 353 and 354
	6b	Any changes to trial outcomes after the trial commenced, with reasons	N/A
Sample size	7a	How sample size was determined	Page 3 Lines 283-290
	7b	When applicable, explanation of any interim analyses and stopping guidelines	N/A
Randomisation:			
Sequence	8a	Method used to generate the random allocation sequence	page 4 lines 379-384
generation	8b	Type of randomisation; details of any restriction (such as blocking and block size)	Page 4 lines 379-382
Allocation	9	Mechanism used to implement the random allocation sequence (such as sequentially numbered containers),	

concealment mechanism		describing any steps taken to conceal the sequence until interventions were assigned	page 4 379-380
Implementation	10	Who generated the random allocation sequence, who enrolled participants, and who assigned participants to interventions	page 4 378-382<br>page 4 359-388
Blinding	11a	If done, who was blinded after assignment to interventions (for example, participants, care providers, those assessing outcomes) and how	N/A
	11b	If relevant, description of the similarity of interventions	N/A
Statistical methods	12a	Statistical methods used to compare groups for primary and secondary outcomes	page 5 lines 500-582
	12b	Methods for additional analyses, such as subgroup analyses and adjusted analyses	page 5 560-563, , 576-582
**Results**			
Participant flow (a diagram is strongly recommended)	13a	For each group, the numbers of participants who were randomly assigned, received intended treatment, and were analysed for the primary outcome	figure 1 page 5,
	13b	For each group, losses and exclusions after randomisation, together with reasons	page 5, lines 500-507
Recruitment	14a	Dates defining the periods of recruitment and follow-up	page 4 line 393
	14b	Why the trial ended or was stopped	sample size was achieved
Baseline data	15	A table showing baseline demographic and clinical characteristics for each group	page 7, table 1
Numbers analysed	16	For each group, number of participants (denominator) included in each analysis and whether the analysis was by original assigned groups	page 7 table 1 and 2
Outcomes and estimation	17a	For each primary and secondary outcome, results for each group, and the estimated effect size and its precision (such as 95% confidence interval)	Page 6 line 627-630, 633-639, 643-650, table 1 page 7, table 4 page 9
	17b	For binary outcomes, presentation of both absolute and relative effect sizes is recommended	
Ancillary analyses	18	Results of any other analyses performed, including subgroup analyses and adjusted analyses, distinguishing	N/A

		pre-specified from exploratory	
Harms	19	All important harms or unintended effects in each group (for specific guidance see CONSORT for harms)	N/A
**Discussion**			
Limitations	20	Trial limitations, addressing sources of potential bias, imprecision, and, if relevant, multiplicity of analyses	page 11 lines 1232-1239
Generalisability	21	Generalisability (external validity, applicability) of the trial findings	
Interpretation	22	Interpretation consistent with results, balancing benefits and harms, and considering other relevant evidence	page 8,9,10
**Other information**			
Registration	23	Registration number and name of trial registry	N/A
Protocol	24	Where the full trial protocol can be accessed, if available	Available on request
Funding	25	Sources of funding and other support (such as supply of drugs), role of funders	N/A
